# Unsupervised online reaction time test improves estimation of cognitive function in a large sample of cognitively‐asymptomatic older adults

**DOI:** 10.1002/alz.091033

**Published:** 2025-01-09

**Authors:** Xinyi Wang, Aidan Bindoff, Rebecca J St George, Eddy Roccati, Larissa Bartlett, Katherine Lawler, Alastair J Noyce, Son Tran, Anna E King, James C Vickers, Quan Bai, Jane E Alty

**Affiliations:** ^1^ Wicking Dementia Research and Education Centre, University of Tasmania, Hobart, TAS Australia; ^2^ School of Psychological Sciences, University of Tasmania, Hobart, TAS Australia; ^3^ La Trobe University, Bundoora, VIC Australia; ^4^ Queen Mary University of London, London, England UK; ^5^ School of Information and Communication Technology, University of Tasmania, Hobart, TAS Australia; ^6^ Royal Hobart Hospital, Hobart, TAS Australia; ^7^ School of Medicine, University of Tasmania, Hobart, TAS Australia

## Abstract

**Background:**

Finding low‐cost, accessible methods to detect people at risk of preclinical Alzheimer’s disease (AD) is a research priority for neuroprotective drug development and clinical trials. Sub‐clinical asymptomatic decline in episodic memory is a proxy measure of preclinical AD and there is emerging evidence that analysis of motor patterns using laboratory apparatus may detect this stage. There has been little investigation of whether self‐administrated simple hand motor tests using personal computers in an unsupervised environment can detect preclinical AD. This study evaluated how home‐based reaction time tests predict cognitive performance in a sample of cognitively asymptomatic older adults.

**Method:**

894 community participants (66.2 +/‐ 7.46 years old, 71.0 % female) who self‐reported an absence of cognitive symptoms were recruited from the ISLAND cohort study. In an unsupervised environment such as their home, they completed a simple reaction time test and a five‐choice reaction time test from the TAS Test website, and validated online cognitive tests (CANTAB) of episodic memory, working memory and executive function. Reaction time was measured in milliseconds. Test failure was defined as a delay of > 2000 milliseconds. We fitted hurdle linear mixed models to examine associations between reaction time, and test failure, with cognitive performance, adjusted for choice (simple or 5‐choice).

**Result:**

Higher episodic and working memory errors, and worse executive function scores were associated with test failure (episodic memory log‐odds = 0.135, p < 0.001, working memory log‐odds = 0.130, p = 0.002, and executive function log‐odds= 0.132, p < 0.001). There was no significant association between cognitive test scores and reaction time on successful trials (episodic memory p = 0.438, working memory and executive function models would not converge).

**Conclusion:**

Test failure in brief self‐administered online reaction time tests were associated with cognitive test performance across three domains, in a cognitively asymptomatic older cohort. This provides a potential low‐cost and brief home‐based method for risk stratification of enriched cohorts for further assessment.

